# Therapeutic Potential of Innate Lymphoid Cells for Multiple Myeloma Therapy

**DOI:** 10.3390/cancers13194806

**Published:** 2021-09-26

**Authors:** Aneta Szudy-Szczyrek, Sean Ahern, Magdalena Kozioł, Daria Majowicz, Michał Szczyrek, Janusz Krawczyk, Marek Hus

**Affiliations:** 1 Chair and Department of Haematooncology and Bone Marrow Transplantation, Medical University of Lublin, 20-081 Lublin, Poland; magkoziol@op.pl (M.K.); daria.majowicz@umlub.pl (D.M.); 2 Department of Haematology, University Hospital Galway, H91 TK33 Galway, Ireland; sean.ahern@galwayclinic.com (S.A.); janusz.krawczyk@nuigalway.ie (J.K.); 3 National University of Ireland, H91 TK33 Galway, Ireland; 4 Chair and Department of Pneumonology, Oncology and Allergology, Medical University of Lublin, 20-954 Lublin, Poland; michal.szczyrek@umlub.pl

**Keywords:** innate lymphoid cells, multiple myeloma, therapy, NK cells, cell therapy, bone marrow, microenvironment

## Abstract

**Simple Summary:**

Multiple myeloma (MM) is the second most common haematological malignancy. Despite huge progress associated with the introduction of new antimyeloma drugs, MM remains an incurable disease. In this review, we discuss the role of the innate lymphoid system, its role in the pathogenesis of the disease, and the mechanisms by which innate lymphoid cells (ILC) can theoretically achieve therapeutic benefit in MM treatment.

**Abstract:**

Innate lymphoid cells (ILCs) are a recently identified family of lymphocyte-like cells lacking a specific antigen receptor. They are part of the innate immune system. They play a key role in tissue homeostasis and also control inflammatory and neoplastic processes. In response to environmental stimuli, ILCs change their phenotype and functions, and influence the activity of other cells in the microenvironment. ILC dysfunction can lead to a wide variety of diseases, including cancer. ILC can be divided into three subgroups: ILC Group 1, comprising NK cells and ILC1; Group 2, including ILC2 alone; and Group 3, containing Lymphoid Tissue inducers (LTi) and ILC3 cells. While Group 1 ILCs mainly exert antitumour activity, Group 2 and Group 3 ILCs are protumorigenic in nature. A growing body of preclinical and clinical data support the role of ILCs in the pathogenesis of multiple myeloma (MM). Therefore, targeting ILCs may be of clinical benefit. In this manuscript, we review the available data on the role of ILCs in MM immunology and therapy.

## 1. Introduction

Multiple myeloma (MM) is the second most common haematological malignancy. Its incidence worldwide is approximately 160,000 cases per year. The mean age of onset is 70 years [1]. The spectrum of clinical symptoms is variable. Initial symptoms are usually mild and non-specific, including low-grade fever, generalised weakness, weight loss and recurrent infections. As the disease progresses, patients develop disseminated bone lesions, pathological fractures, bone marrow failure and renal injury. Despite the enormous therapeutic progress thanks to the introduction of proteasome inhibitors (PIs), immunomodulatory drugs (IMIDs) and targeted therapy, in the form of monoclonal antibodies, MM remains an incurable disease. Relapse is a common occurrence, even after complete remission (CR) has been achieved. The median survival is approximately 6 years [2,3,4].

The clonal evolution of MM cells, changes in the bone marrow microenvironment (BMME) and complex interactions between cancer cells and the bone marrow niche mediate observed resistance to current therapies. Current therapeutic goals in MM include identifying myeloma stem cells and their unique drug resistance features, understanding the oligoclonal evolution of MM cells as well as changes in the immune and non-immune microenvironment [5,6]. In this review, we discuss the role of the innate lymphoid system in the pathogenesis and therapy of MM.

## 2. Innate Lymphoid Cells

Innate lymphoid cells (ILCs) are characterised by three main features: lymphoid morphology, lack of specific antigen receptors and lack of phenotypic markers of myeloid cells and dendritic cells (DCs) [7,8]. They are the innate counterparts of T lymphocytes and constitute a heterogeneous group of cells, including NK cells as well as non-cytotoxic cells. It is hypothesised that they arise from the Common Lymphoid Progenitor (CLP).

ILCs, based on phenotypic and functional characteristics, are divided into three subgroups: ILC Group 1, which includes NK and ILC1 cells; Group 2, which contains only ILC2 cells; and Group 3 ILCs, which includes Lymphoid Tissue inducer (LTi) and ILC3 cells. While ILC1s, ILC2s and ILC3s mirror the function of CD4+ Th1, Th2 and Th17 T helper cells, respectively, NK cells mirror the function of CD8+ cytotoxic T cells [9,10]. ILCs express effector cytokines typically associated with T helper cells and are therefore expected to play a central role in coordinating immune responses.

ILC1 is characterised by the expression of T-bet (a transcription factor that controls IFN-γ) and the production of IFN-γ, which is essential in immunity against intracellular pathogens, viruses and cancer [11,12].

ILC2s are a heterogeneous population of cells consisting of distinct subsets with different tissue localisation and cytokine reactivity. ILC2s require Gata3 for differentiation and survival [13]. They respond to epithelial-derived alarmins, i.e., IL-25, IL-33 and thymic stromal lymphopoietin (TSLP). When activated by IL-25 and IL-33, ILC2s produce cytokines such as IL-5 and IL-13, which are essential in response to large extracellular parasites and allergens. IL-5 activates cells involved in type 2 immunity and mediates eosinophil recruitment and activation, as well as secretion of pro-inflammatory IL-4 [14,15].

ILC3s are defined by the expression of retinoic-acid-receptor-related orphan nuclear receptor γ (RORγt) [16]. Activation of ILC3s, like that of ILC2s, is regulated by multiple soluble factors, including cytokines, neuronal factors, metabolites and inflammatory mediators. The main regulatory cytokines include IL-1α, IL-1β, IL-2, IL-7, IL-23, tumour necrosis factor-like cytokine 1A (TL1A), stem cell factor (SCF) and thymic stromal lymphopoietin (TSLP), which are secreted by T lymphocytes, myeloid, epithelial and stromal cells. IL-7 is a necessary factor in the formation of ILC3s. ILC3s secrete IL-22 and IL-17, which are extremely important for resistance to bacterial and fungal infections. They also produce IL-2, which is critical for maintaining T cells in immune balance [17,18].

ILCs begin to function in the early period of immune system formation, during foetal development, as LTi cells [19]. These cells induce the development of secondary lymphoid organs and are required to develop lymph nodes and Peyer’s patches. LTi actively migrate to lymphoid organs and promote the development of lymphoid tissue. They affect mesenchymal stromal cells to produce factors that activate chemotaxis of hematopoietic cells to the developing lymphoid structures [7,19,20]. The interaction between the ILC and the non-hematopoietic microenvironment is an important aspect of the lifelong function of ILCs. It involves the activation of stromal cells for the recruitment, retention, activation and regeneration of lymphocytes, as well as the activation of defence and anti-apoptotic pathways. ILCs play a key role in the communication of lymphoid cells with non-hematopoietic cells and in the spatial organisation of immunity [7].

ILCs are highly reactive and act early in the immune response. They are activated by environmental signals, microbial compounds, cytokines, alarmins, hormones, neuropeptides, and eicosanoids. They act like “congenital” or memory T cells such as NKT cells and γ subsets [21]. Later in the immune response, several days after the onset, active ILC and T cells interact. ILCs activate antigen-specific T cells by expressing class II major histocompatibility complex (MHC) molecules and modifying antigens. T lymphocytes, in turn, produce IL-2, which promotes the activity of ILCs. Both cell types are subject to feedback loops that enhance their responses [19].

## 3. Innate Lymphoid Cells in the Multiple Myeloma Microenvironment

**Figure 1:**
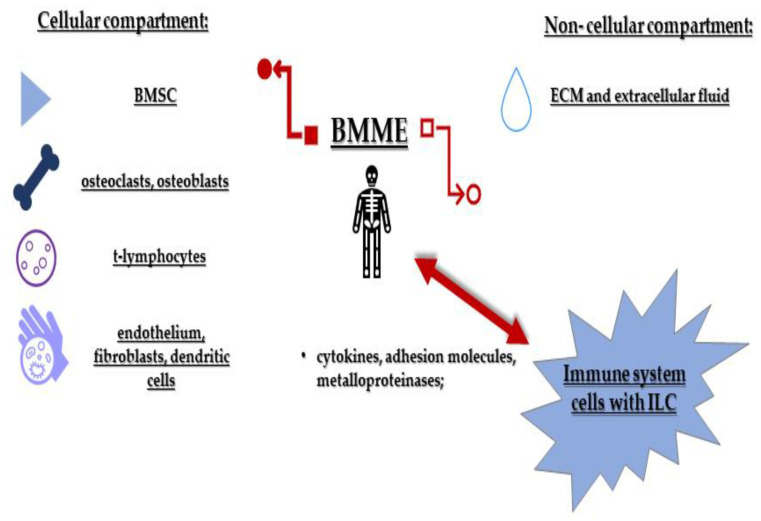
Innate lymphoid cells in the multiple myeloma microenvironment. The BMME consists of two components: cellular and non-cellular compartments, that interact with each other. ILCs play a key role in controlling tissue homeostasis through interactions with the BMME. ECM—extracellular matrix; ILC—innate lymphoid cells; BMME—bone marrow microenvironment; BMSC—bone marrow stromal cells.

The bone marrow microenvironment (BMME) consists of two components: cellular and non-cellular. The cellular component includes bone marrow stromal cells (BMSC), endothelium, fibroblasts, osteoclasts and osteoblasts, T-lymphocytes, and dendritic cells, and the non-cellular component is formed by an extracellular matrix (ECM) and a fluid environment that contains cytokines, chemokines and growth factors. The physiological role of the bone marrow stroma is to regulate and support the growth and differentiation of hematopoietic cells. Therefore, the BMME undergoes significant alterations during the onset and progression of MM. In the pathogenesis of MM, interactions between microenvironment cells (in particular, endothelial cells and MSCs) and the neoplastic clone occur through direct interactions of surface adhesion and receptor molecules and by mediators released by these cells. These interactions enable the survival, proliferation and differentiation of MM cells (Figure 1) [22,23,24].

Early reports of tumour-infiltrating leukocytes suggest a functional relationship between immune system cells (ISs) and cancer [25]. Initially, these infiltrating ISs were thought to have anticancer properties. However, recent discoveries also point to a role in promoting cancer progression. Increasingly, it is appreciated that interactions between tumour-initiating cells and ISs, including ILCs and cells in the microenvironment, play a key role in tumour formation and metastasis. Both neoplastic cells and ISs are mediated by cytokines, adhesion molecules, and metalloproteinases [26]. The first response to tumour formation is the mobilisation of ISs and activation of cytotoxic mechanisms through the production of cytokines to induce apoptosis of neoplastic cells [27]. When proliferating neoplastic cells gain the advantage over ISs, ILCs begin to produce pro-neoplastic growth factors [28,29].

It is also worth mentioning that interactions between ISs and other microenvironment components influence the course of a neoplasm [30]. It has been reported that the ECM can play both a supporting and suppressing role in the adaptive immune response, providing migration pathways that allow T cells to invade tissue or directly inhibit T cell proliferation [31]. Lymphatic vessels, which, thanks to increased angiogenesis, are involved in the supply of nutrients to the neoplastic tissue, may serve as migration routes for immune cells [32]. Interaction between ICs and stromal cells is also recognised [33].

ILCs are largely tissue-resident cells and are deeply integrated into those tissues in which they reside. They play a crucial role in homeostasis because of the speed with which they react and their on-site presence in normal, healthy tissues [7,30]. ILCs are involved in tissue regeneration processes not conventionally associated with the immune system. It has been proven that they are capable of local migration, especially during inflammation [31]. Another unique feature is their “plasticity”, the ability to differentiate from one subtype to another depending on the signals received from the environment [32,33,34]. In the context of cancer, this plasticity may alter the characteristics of ILC with antitumour or tumour-promoting properties [35].

## 4. Innate Lymphoid Cells in the Prevention of Multiple Myeloma

### 4.1. The Role of NK Cells

Within the entire ILC family, NK cells play a particularly key role in cancer surveillance. NK cells are large granular CD56+ CD3− lymphocytes, which are a key subset of the innate immune system [28,36]. They exhibit “spontaneous” cytotoxicity independent of costimulatory signals or gene rearrangement events, which makes them functionally unique to B and T lymphocytes [37,38]. Moreover, they divide into two distinct, mature human cell populations that perform complementary functions.

CD56dim NK cells constitute approximately 90% of the peripheral blood population and are capable of targeted and potent cytotoxicity. Conversely, CD56bright NK cells are found mainly in secondary lymphoid tissue and sites of the ongoing inflammatory process and are capable of intense production of cytokines in the developing immune response. NK cells kill target cells directly by releasing cytotoxic granules containing granzymes and perforin, or indirectly through activation of TNF ligand, TNF apoptosis-inducing ligand (TRAIL) and Fas ligand (FasL) receptors. In addition, they also secrete a wide range of cytokines and chemokines, of which IFN-γ is known for its potent anticancer properties [36].

MM cells have been shown to express CD1d, which belongs to the CD1 family of antigen-presenting molecules. The structure of CD1d molecules resembles the antigens of the major histocompatibility complex (MHC). They consist of the α1, α2, and α3 chains associated with β2 microglobulin. CD1 molecules are monomorphic themselves but can attach to various lipid-based antigens due to the two hydrophobic pockets in their structure. The presence of CD1d on the surface of MM cells makes them vulnerable to NK cell attack [39,40,41,42]. During MM evolution, a progressive reduction in CD1d expression in vivo is observed. Initially, Cd1d surface expression is lost but persists in the cytoplasm. In more advanced and terminal stages, such as plasmacytoma cutis, loss of expression is associated with inhibition at the level of transcription [43].

### 4.2. The Role of ILC1s

As already mentioned, ILC1s produce IFN-γ, which is believed to protect against cancer. Interferons are a group of pleiotropic proteins that are important both in innate and acquired immunity. IFN-γ is the only member of the type II class of interferons. In addition to responding to infections, it plays a role in inhibiting neogenesis. In terms of structure, it is a homodimer composed of two antiparallel polypeptide units. A single molecule can therefore attach to two receptors, thereby enhancing the immune response. Furthermore, the IFN-γ response can lead to cross-communication with interferon α and β receptors. IFN-γ is involved in the control of transcription and translation of genes involved in the regulation of the cell cycle, apoptosis, intercellular interaction, and antigen presentation [44,45,46].

The antiproliferative effect of IFN-γ is based on the activation of STAT 1 in the tumour cell via the regulation of cyclin-dependent kinase inhibitor 1 (p21) [47]. Induction of apoptosis by IFN-γ is associated with an increase in the expression of caspases: 1, 3 and 8 [48]. However, this is not the only mechanism for inducing programmed cell death. IFN-γ has been observed to increase the expression of Fas and FasL on the surface of cancer cells [49]. Xu et al. demonstrated that IFN-γ-induced Fas overexpression occurred in STAT1-positive cells and was absent in STAT1-deficient cells. Anti-Fas antibody, however, was able to induce apoptosis in both cell lines. The researchers suggested that STAT1 enhances the expression of Fas and FasL, but does not participate in Fas-triggered proteolysis [49]. IFN-γ also increases MHC expression on the cell surface, which is associated with facilitating the recognition of cancer cells by the immune system [44]. Additionally, IFN-γ contributes to an increase in the number of Th1 cells while reducing the Th2 population [50].

IFN-γ exhibits inhibition of MM cell proliferative activity. This effect has been demonstrated both on IL-6-dependent myeloma cell lines and on MM cells freshly obtained from bone marrow. It has been suggested that the antiproliferative activity of IFN-γ is mainly due to the inhibition of IL-6, the key growth factor of MM. Inhibition of IL-6 can occur at various levels: downregulation of the IL-6 receptor has been reported, and blockade of the IL-6 signalling pathway by interaction with cytoplasmic proteins such as p91 has been suggested. Studies by Palumbo et al. have shown that IFN-γ inhibits the development of MM to the same extent as dexamethasone [51,52]. Martins et al. observed higher IFN-γ concentration in preparations collected before autologous stem cell transplant (ASCT) in patients who achieved complete remission (CR) 3 months after ASCT compared to those with very good partial response (VGPR) [53].

However, it has been reported that IFN-γ directly increases the expression of B-cell CLL/lymphoma 6 (BCL6), one of the oncogenic transcriptional regulators in MM cells [54,55]. Ujvari et al. found that IFN-γ strongly induces mRNA and BCL6 protein expression in MM cell lines via the classical STAT1 signalling pathway [56].

## 5. Innate Lymphoid Cells in the Development of Multiple Myeloma

**Figure 2:**
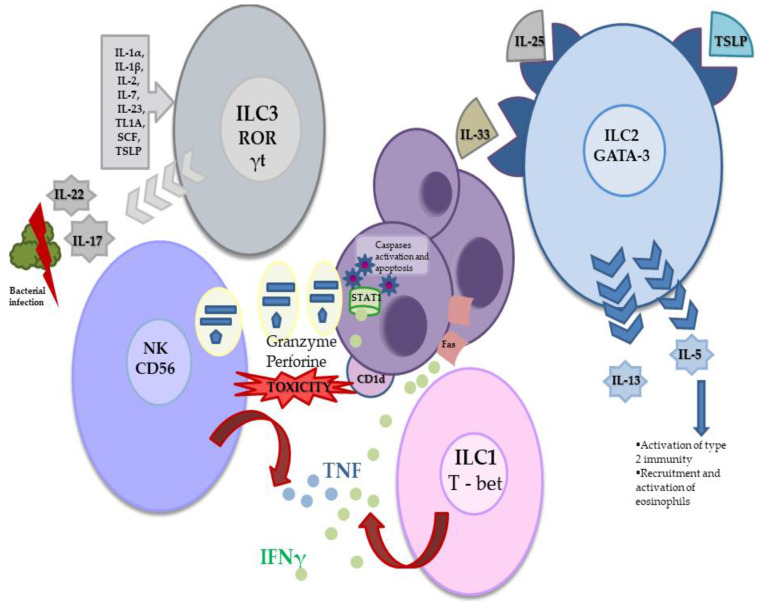
The role of ILCs in MM pathogenesis. RORγt—retinoic-acid-receptor-related orphan nuclear receptor γ; SCF—stem cell factor; TL1A—tumour necrosis factor-like cytokine 1A; TSLP—thymic stromal lymphopoietin.

Neoplastic cells escape from immune surveillance via two basic mechanisms: immunoediting of neoplastic cells and suppression of immune functions [57]. Both of these phenomena are observed in MM (Figure 2).

### 5.1. NK Cell Dysfunction

Previous studies have confirmed an increased number of CD56+ CD3− NK cells in the bone marrow and blood in newly diagnosed MM patients and with monoclonal gammopathy of undetermined significance (MGUS). Interestingly, patients with a higher number of NK cells at the time of diagnosis had a worse prognosis [36]. The increased number of NK cells has been attributed to ineffective activation of the immune system to control MM cell expansion [36,37].

We currently know that NK cell activity in MM patients is profoundly impaired [36,37,56,58]. The mere presence of an excessive amount of immunoglobulins in the serum affects the functioning of NK cells. Researchers have observed diminished ADCC function and a decrease in the number of cytolytic granules with the appearance of intracellular vacuoles, which corresponded to a decrease in NK cell cytotoxicity [59,60]. It has been shown that monomeric IgG (especially subclasses 1 and 3), as well as monoclonal IgA and IgG proteins from MM patients, exert a depressive effect on NK cell function [61,62,63,64].

#### 5.1.1. Humoral Mechanism

The cytokine axis, important for the activation, proliferation and function of NK cells, is disrupted in MM [65,66].

MM cells together with CD4+ CD25+ regulatory T cells, the percentage of which is significantly increased in MM, secrete transforming growth factor β (TGF-β) [67,68]. In NK cells, stimulation of TGF-β induces phosphorylation of SMAD3, leading to the inhibition of IFN-γ production mediated by CD16 and ADCC [69]. TGF-β also reduces the ability of NK cells to respond to pro-inflammatory monokines such as IL-12 and IL-15 [70].

In addition, other cytokines abundant in MM, such as IL-6 and IL-10, contribute to NK cell dysfunction. IL-6-producing tumours have been shown to interfere with the cytotoxicity of NK cells [70]. IL-10 antagonises the production of pro-inflammatory IFN-γ and TNF-α [71,72] and promotes the development of NK-resistant tumour phenotypes [73].

Other soluble factors are known to suppress NK-mediated antimyeloma capabilities. The expression of cyclooxygenase-2 (COX-2) on MM cells leads to the production of prostaglandin E2 (PGE2) [58,71]. PGE2 increases the level of cyclic adenosine monophosphate (cAMP) and inhibits activating signals transduced by NCR, NKG2D, and CD16. This results in the inhibition of NK cell cytotoxicity, cytokine synthesis and release [70,74]. The high concentration of soluble IL-2 receptors observed in the serum of patients with MM may interfere with the activation of NK cells mediated through IL-2 by T lymphocytes [75]. Indoleamine 2,3-dioxygenase (IDO) promotes neoplastic cell immune escape through antigen-presenting cells via enzymatic degradation of L-tryptophan [76]. It has been shown that IDO-mediated immunosuppression also involves NK cells via L-kynurenine (Kyn), a L-tryptophan degradation product impairing NKp46/NKG2D-specific lysis [77]. Interaction between CD28 on MM cells and CD80/86 affects IDO synthesis by stromal dendritic cells, in agreement with the observation that CD28 expression on MM cells correlates with poor prognosis [78].

Humoral factors lead to disturbances in intracellular signalling pathways. For example, constitutive or cytokine-induced activation of signal transducer and activator of transcription 3 (STAT3) promotes the multiplication and growth of MM cells [79,80].

#### 5.1.2. Signalling Mechanism

Diminished NK cell activity may result from disturbed receptor–ligand interactions. For example, NK cells require the activating of DNAM-1, NKG2D and/or NKp46 receptors for the cell-killing process [81].

In the course of the progression of MGUS to advanced MM, the surface expression of MICA—the ligand for NKG2D on MM cells—is lost, and MICA is secreted in a soluble form [82,83,84]. It has been shown that activation of STAT3 inhibits MICA transcription and expression and that MM cells have high levels of ERp5, a disulphide isomerase that promotes MICA excretion [83,84,85,86].

The identification of myeloma cells by NK cells is downregulated by MHC class I expression on MM cells. Class I MHC expression is negligible in early MM but high in advanced disease [83]. An evolution of MHC class I expression in progression from MGUS to MM and plasma cell leukaemia has been observed. This change has been correlated with an increase in levels of soluble MHC class I and β2-microglobulin [87]. Higher soluble class I MHC and β2-microglobulin correlated with shorter survival and poor prognosis [87]. This is especially important for the suppression of NK cell cytotoxicity.

Moreover, MM cells have been shown to express PD-L1, a programmed death receptor 1 (PD-1) ligand [88,89]. PD-1 is expressed on T and NK cells in MM, and the PD-1/PD-L1 interaction can suppress both acquired and innate immunity [89].

The expression of DNAM-1 on NK cells in patients with active MM is also reduced compared to healthy controls and patients in remission [80,90]. In addition, lower expression of NKG2D, 2B4 (CD244) and CD16 was also confirmed in the course of MM [91,92].

Another phenomenon described in the course of MM is Fas downregulation [93] and loss of function of the Fas antigen, which makes these cells resistant to lysis induced by a mechanism related to the Fas/FasL signalling pathway [94].

### 5.2. The Role of ILC1s

Due to their relatively recent discovery and the lack of specific markers for identifying non-NK cells, little is known about the role of ILC1s in MM development and progression. However, recent observations imply an urgent need for research.

In patients with plasma cell dyscrasias, an increase in the percentage of ILC1s is observed in the bone marrow. While the ability of ILC1s to secrete line-specific cytokines (IFN-γ) was preserved in patients with MGUS, it was significantly reduced in patients with asymptomatic MM [95].

Interestingly, human ILC1 subsets have been shown to express high levels of Ikzf3 (Ajolos), a transcription factor involved in B cell differentiation, a known immunomodulatory drug (IMiDs) target. Pomalidomide—a drug from the IMiDs group used in the treatment of MM—restores the secretion of IFN-γ by ILC1s [96].

### 5.3. The Role of ILC2s

Due to the secretion of cytokines involved in promoting tumour growth and blocking antitumour immunity in the microenvironment, ILC2 is widely recognised as a cell subtype with protumour properties [96]. It has been shown that the ILC2/IL-13/myeloid-derived suppressor target (MDSC) axis contributes to creating the immunosuppressive microenvironment of acute promyelocytic leukaemia and many lithium tumours [97,98,99]. Undoubtedly, a desirable direction of research would be to also investigate the role of the ILC2/IL-13/MDSC axis in the pathogenesis of MM. So far, data on the importance of ILC2 in the context of MM development are limited.

In patients with plasma cell dyscrasias, a decrease in the number of ILC2s in the bone marrow was noted with a simultaneous increase in the circulating subset. In patients with MGUS, ILC2s demonstrated the ability to secrete IL13, which was not observed in patients with asymptomatic MM [96].

Guillerey et al. investigated the role of ILC2s in the bone marrow, and IL-33 stimulated ILC2s in MM. They found that the growth of MM was associated with phenotypic and functional changes in ILC2, increased expression of maturation markers, and decreased ability to produce cytokines upon stimulation with IL-2 and IL-33. The role of ILC2s in the regulation of MM development and growth was not confirmed in the study. Nevertheless, researchers observed that IL-33 induces the circulating inflammatory population KLRG1h and ILC2s and inhibits type 1 immunity against MM [100].

ILC2s express the PD-1 immune checkpoint whose ligand, PD-L1, is highly expressed in the MM microenvironment [101,102]. It has been suggested that PD-1/PD-L1 interactions may lead to ILC2 depletion in MM.

### 5.4. The Role of ILC3s

The protumour activity of ILC3s has been emphasised in many types of cancer due to the release of cytokines involved in the pathogenesis of inflammatory diseases, i.e., IL-17, IL-22, and IL-23 [103,104]. There are few data on the role of ILC3s in MM biology. Theoretically, however, ILC3s may be crucial in the development and progression of MM. Patients with active MM have higher concentrations of IL-17, IL-22, and IL-23 in the blood and bone marrow compared to the control group. IL-22 has been shown to increase with disease activity, in conjunction with IL-1β, confirming some inflammatory elements in the disease pathogenesis [105,106]. IL-17 promotes MM cell growth and colony formation through the IL-17 receptor, adhesion to bone marrow stromal cells (BMSC) as well as enhanced in vivo growth in a mouse–human MM xenograft model. IL-17A promotes the development of MM but also inhibits immune functions in the microenvironment [107]. High levels of IL-17 are associated with a poor prognosis [108].

ILC1s are characterised by the expression of T-bet and the production of IFN-γ. ILC2s require Gata3 for differentiation and response to IL-25, IL-33 and TSLP. After activation by IL-25 and IL-33, ILC2s produce cytokines such as IL-5 and IL-13. IL-5 activates bone marrow cells involved in type 2 immunity and mediates the recruitment and activation of eosinophils. The expression of RORγt characterises ILC3s. The activation of ILC3s is regulated by many regulatory cytokines such as IL-1α, IL-1β, IL-2, IL-7, IL-23, TL1A, SCF and TSLP. ILC3s secrete IL-22 and IL-17, which are extremely important for resistance to bacterial and fungal infections. NK cells are large granular CD56+ CD3− lymphocytes, which kill target cells directly by releasing cytotoxic granules containing granzymes and perforin, or indirectly through TNF ligand and Fas ligand receptors. They also secrete cytokines and chemokines, of which IFN-γ is known for its potent anticancer properties. The antiproliferative effect of IFN-γ is based on the activation of STAT 1. Induction of apoptosis by IFN-γ is associated with an increase in the expression of caspases: 1, 3 and 8. MM cells have been shown to express CD1d. The presence of CD1d on the surface of MM cells makes them vulnerable to NK cell attack.

## 6. Targeting Innate Lymphoid Cells for Multiple Myeloma Immunotherapy

The fifth and most recently FDA approved CAR-T cell therapy is idecabtagene vicleucel, trade name Abecma, with a CAR against B-cell maturation antigen (BCMA), for the treatment of adult patients with relapsed or refractory MM after four or more prior lines of therapy, including an immunomodulatory agent, a proteasome inhibitor, and an anti-CD38 monoclonal antibody [109]. At the time of writing, there are no regulator-approved ILC cell therapies for any indication. The most likely first approval will be a CAR-NK cell therapy, possibly for haematological malignancy, based on current research emphasis [110].

### 6.1. Non-NK ILC Cell Therapy for MM

Non-NK ILC’s relatively recent discovery with attendant ongoing challenges in characterisation [111], confounding predilection for plasticity [112], and their perception as being primarily non-cytotoxic action makes them less likely to appear in the first phase of ILC cell therapies [113]. Nonetheless, we explore some possible avenues for ILC cell therapy for MM.

NK-like ILC1s (ILC1s expressing high levels of granzyme A and CD160) can be selected for antitumour cytotoxic potential [114], and their TRAIL-mediated cytotoxicity is calibrated by activating receptor NKp46 [115]. TRAIL is a potent inducer of apoptosis in MM [116]. These data support further exploration of selected and potentiated tumoricidal ILC1s as an anti-MM cell therapy candidate, but any advantages over conventional NK approaches are not apparent. The recent discovery of cytotoxic ILC3s offers another potential candidate for cytotoxic non-NK ILC cell therapy development. These CD94+ cells appear to mirror cytotoxic CD4+ T cells (CD4+ CTLs). They are CD16+, do not express KIR and are induced to cytotoxicity by IL-12 rather than IL-2 in the case of CD4+ CTLs [117].

Interleukins may provide a pathway to therapeutic manipulation of ILCs present in MM patients, although this has not been achieved yet. Sustained IL-33 production local to tumours was found to induce massive proliferation of intratumoral ILC2s, which stopped tumour growth [118]. This effect was not recreated in an MM model where single systemic IL-33 administration induced a circulating ILC2 population but inhibited type 1 innate antitumour immunity. The same study found that IL-12/IL-18 administration had a therapeutic effect likely via IFN-γ production by innate immune cells [111].

#### ILCs in Graft-Versus-Host Disease

The utility of allogeneic stem cell transplantation (allo-SCT) in MM is controversial and limited by graft-vs.-host disease (GvHD). However, allo-SCT is thus far the only potentially curative treatment approach [119,120]. Here, in the context of MM, we outline an evidence base that suggests real potential for the use of ILCs in GvHD. Notably, GvHD is also a concern in CAR-T cell therapies [121], which are mentioned later.

Faster recovery of ILC counts, higher ILC expression of gut and skin chemokines and higher donor-derived ILC3 counts are associated with less GvHD post allo-HSCT [122]. IL-22 secretion by recipient NKp46-ILC3s in the intestine and thymus was found to prevent GvHD by enhancing intestinal stem cell (ISC) function, thymic regeneration and T-cell reconstitution in post allo-HSCT GvHD models [123,124,125]. CD39+/CD73+ ILC3s secrete adenosine to create a T-cell suppressive microenvironment. These cells are depleted in GvHD, and their loss is a putative contributor to GvHD. Of note, this purinergic pathway of effector cell suppression is discussed below in the context of CD38 immune cell depletion.

ILC2s also support ISCs [126]. Gut ILC2s are poorly reconstituted post allo-HCST, and in a mouse model, co-transfusion of ex vivo expanded IL-33-activated ILC2s with T-cells prevented GvHD. The translatability of this ILC2 infusion to prevent GvHD remains to be determined [127]. Along with the evidence from ILC3 populations, this study points to a potential prophylactic and/or therapeutic role for donor-derived ex vivo expanded selected and activated ILC2/3 adoptive cell therapy in MM patients post allo-HSCT or CAR-T cell therapy, in which trafficking to skin and gut may mitigate any adverse effects on graft-vs.-myeloma.

### 6.2. NK ILC Cell Therapy for MM

#### 6.2.1. NK Cells during Daratumumab Therapy

Daratumumab (Dara) is an anti-CD38 monoclonal antibody that potently directs Fc-dependent complement-dependent cytotoxicity (CDC) and antibody-dependent cellular phagocytosis (ADCP), but most of its effect is ascribed to antibody-dependent cellular cytotoxicity (ADCC) of CD38^+^ cell sets. As well as many MM clones, most NK cells are CD38^+^. Dara treatment induces NK cell fratricide, which rapidly depletes the NK cell compartment, with only CD38^−/low^ subsets remaining following exposure, which in turn can lead to higher susceptibility to infections [128]. Although the data from two studies (GEN501 and SIRIUS) did not confirm a relationship between NK-cell count reduction and efficacy of daratumumab or safety profile, the exact link between immune impairment and infectious complications during Dara therapy is still under debate [129]. The question logically arises as to whether supplementing Dara with NK cells could add its benefits.

All-trans retinoic acid and interferon-α have been found to upregulate CD38 expression in MM cells. This strategy was combined with applying an anti-CD38 nanobody to NK-92 cells in an ex vivo experiment to shift the balance of CD38 expression between effector and target cells, with findings of proportional promotion of MM cell death by shifting the target away from NK cells [130].

Aggregate ADCC by NK cells is likely not diminished by the same factor as the loss in NK counts post Dara exposure. Ex vivo studies indicate that the remaining CD38^−/low^ subsets are highly activated through increased expression of genes associated with the immune response [131,132]. Pretreatment of ex vivo expanded NK cells (eNKs) with Dara to select for CD38^−/low^ populations led to increased cytotoxicity against preclinical models of MM in comparison to Dara non-pretreated NK cells [133]. eNKs from Dara-treated patients were more proliferative than those from MM patients not treated with Dara, or those from healthy patients. These eNKs effectively killed MM cells in a mouse model, and their effect was augmented by Dara co-treatment. Phenotypic drift to the inclusion of CD38^+^ NK cells occurred on expansion with anti-CD38 F(ab)_2_ pretreatment of these cells protecting against Dara induced fratricide. The authors suggest autologous transplant of anti-CD38 F(ab)_2_ pretreated eNK to augment ADCC in Dara-treated patients [128]. 

Another study developed the paradigm of allogeneic transplant of CRISPR/Cas9 CD38 gene knockout (CD38^KO^) NK cells using a mouse model. These healthy donor-derived ex vivo expanded CD38^KO^ NK cells were resistant to fratricide and found to have particularly strong efficacy against CD38^low^ MM cell lines, one such line being from a patient who relapsed while on Dara, providing initial evidence for further investigation as supplements in Dara-treated patients [134]. A similar CD38- NK cell batch, selected for CD16 polymorphism, was prepared by use of Dara pretreatment in a cost and time-effective, simple protocol with robust preclinical efficacy results [133]. 

The KHYG1 NK cell line derived from a patient with an NK cell leukaemia is CD38low and can be electroporated to transiently express a CD16 receptor variant encoding the F158V polymorphism (CD16F158V). This mechanism augments Dara activity by supporting ADCC through the non-cleavable CD16 variant. Its particular advantage is the scalability of manufacturing and off-the-shelf availability [135]. FcεRIγ-deficient NK (g-NK) cells are a rare subset derived from CMV seropositive individuals, endogenously CD38low and SLAMF7low. Recent data show that compared to conventional NK cells, ex vivo expanded, unmodified g-NK cells, in combination with Dara, performed excellently in cytotoxicity and persistence assays [136].

The low NK counts may result from the killing of CD38+ clones in Dara-exposed individuals and also reduced proliferation of Dara-exposed NK cells. Furthermore, a recent study [131] showed clear downregulation of genes involved in the cell cycle process that occurs in CD38 mAb-exposed NK cells. This further supports the rationale of transplantation of ex vivo expanded CD38low/- NK cells to Dara-treated patients. Of course, in the well-recognised study mentioned above, eNKs from Dara-treated patients were found to be more proliferative than those from MM patients not treated with Dara, or those from healthy patients [128]. However, this finding remains difficult to reconcile with persistent low NK counts in vivo, which favours translatability of the gene expression study.

#### 6.2.2. CAR-NK Cell Therapy

CAR-NK cell therapy offers numerous advantages over CAR-T cell therapy. HLA-unrestricted CAR-NKs are not associated with GvHD and can be manufactured from allogeneic cell sources such as umbilical cord blood, peripheral blood mononuclear cells, induced pluripotent stem cells and immortalised cell lines such as NK92. It is not associated with a high risk of cytokine release syndrome or neurotoxicity as the cytokine profile—IL-1, IL-2, IL-6, IL-10, IL-15, TNF-α and MCP-1 for CAR-Ts, and IFN-γ and GM-CSF for CAR-NKs—is different. Less immune evasion by the tumour takes place, as cancer cell cytotoxicity also occurs via CAR-independent means (CD16, NKG2D, etc.), whereas in CAR-T cells, it occurs via CAR-mediated mechanisms only [137].

Finding a CAR target effective in MM has predominantly focused on known MM surface antigens. A CD38 CAR has been shown to efficiently target MM cells and overcome BMMSC resistance against NK cells [138]. A BCMA CAR [139] is being trialled in humans (see below). CD138 (syndecan-1) was the target of a CAR construct on NK-92 cells that were found to be more effective against MM xenografts than NK92 cells without this modification [140]. CS1 (CD319, CRACC, SLAMF7), the target for Elotuzumab [141], has also shown promise as a CAR-NK target in preclinical models [142]. An NKG2D-CAR is hoped to be effective for late-stage disease, which, through immunoediting, may no longer express classical antigens and thus, be capable of broad escape [143].

##### Clinical Trials

In August 2021, a search of ClinicalTrials.gov (accessed on 26 August 2021) for NK cell therapies in MM revealed 22 registered clinical trials, which are listed in Table 1. A short narrative on four of these trials is included in the text below, with mention of three other trials not included in Table 1.

A 20-participant, phase 1/2 trial ‘Clinical Research of Adoptive BCMA CAR-NK Cells on Relapse/Refractory MM’ (NCT03940833) is the only trial to use a CAR construct. The CAR target here is BCMA, the same antigen that the most recently approved CAR-T cell therapy idecabtagene vicleucel, mentioned above, uses.

Phase 1 trial ‘FT538 in Subjects with Advanced Hematologic Malignancies’ (NCT04614636) uses a modified NK cell in combination with Daratumumab or in combination with Elotuzumab, in separate arms, against MM. The modification used here is a high affinity, non-cleavable 158V CD16 Fc receptor, which facilitates ongoing ADCC unexhausted by the standard activation-induced surface cleavage. This receptor is not specific for MM but, in combination with MM specific mAbs, is hoped to augment their activity. These cells also have CD38 knockout to circumvent mAb-mediated fratricide [144]

Phase 2 trial ‘Umbilical Cord Blood-Derived Natural Killer Cells, Elotuzumab, Lenalidomide, and High Dose Melphalan, Followed by Stem Cell Transplant in Treating Patients With Multiple Myeloma’ (NCT01729091) uses unmodified umbilical cord blood-derived NK cells late in the conditioning regimen before autologous stem cell transplant.

‘Safety Study Looking at the Use of a Natural Killer Cell Line against Hematological Malignancies’ (NCT00990717) is listed on ClinicalTrials.gov (accessed on 26 August 2021) as a phase 1 trial that enrolled eight participants and was completed in 2012. This trial was reported in 2017 and included data on 12 participants in total, 5 of whom had MM as their diagnosis. The team used repeated infusions of NK-92 cells and showed a good safety profile with mild chills and fever on infusion for three patients. They did note the emergence of anti-HLA antibodies, but these were unrelated to the transfusion reaction, and the reaction did not occur on subsequent infusions. Patient numbers were too small to draw significant conclusions from, but one MM patient did achieve a complete response while receiving concomitant standard maintenance therapy with lenalidomide and dexamethasone [145]. 

Two other non-MM clinical trials worth mentioning, as they include non-NK ILCs in their aims, are ‘Precision Immuno-Oncology for Advanced Non-small Cell Lung Cancer Patients With PD-1 ICI Resistance (PIONeeR-BioMarkers (BM) Profiling) (PIONeeR)’ (NCT03493581) and ‘characterisation of Innate Immune System in Patients With Luminal Advanced Breast Cancer (NIKOLE)’ (NCT04370522). PIONeeR is to enrol 450 participants and looks at non-small cell lung cancer patients receiving standard regimens of immune checkpoint inhibitors (ICIs) and hopes to determine an immune profile that correlates with ICI resistance. NIKOLE is to enrol 31 participants and looks at how progressive disease and hormone resistance correlate with a patient’s innate immune cells.

Similar dedicated ILC studies in MM cohorts would be informative. ‘Immune Profiling in Multiple Myeloma’ (NCT04135079) does not state in its aims that it will be looking at ILCs specifically. The impact of targeting ILCs should not be underestimated and is only beginning to come into view [146]. Unravelling the immunology of ILCs in MM is likely to provide better options for the treatment of MM and other malignancies.

### 6.3. Targeting ILCs through Receptors

Beyond broad actions to ameliorate ILCs’ function [147,148], interest has emerged on ILC metabolism as a therapeutic target [149,150]. While these enhancements are not specifically directed towards the treatment of MM, CD38 knockout via CRISPR/Cas9 resulted in higher mitochondrial respiratory activity, with improved persistence in ex vivo expanded peripheral blood NK cells directed against MM cells [134].

NK cell phenotypes change over the disease course of MM, particularly among bone marrow resident NK cells. The trend is towards exhaustion with upregulation of expression of inhibitory ligands [151,152]. Among NKG2A, TIM-3, TIGIT, VISTA, KIRs, PD-1, CTLA4 and LAG-3, KIRs have been promising [153,154,155], but none have emerged as viable interventions in MM patients [156]. This may be due to the multiplicity of inhibitory pathways occurring and due to heterogeneity across, as well as temporally within, patients being trialled. Further research in this area, with a move towards personalisation, has been advocated [156].

ILC subsets also have unique patterns of activating and inhibitory receptors, which may prove helpful as druggable targets [157]. ILC subsets are likely unintended targets of both PD-1 axis and CTLA4 inhibitors [158]. For example, immune checkpoint inhibitor-related colitis can be fatal and is likely related to disrupted ILC3 function. This effect can be prevented with probiotic administration, which inhibits ILC3s [159]. Immune checkpoint inhibitors are not used routinely in MM, but careful targeting [160] should result in clinical benefit without unintended off-tumour effects.

## 7. Conclusions

In this paper, we reviewed the current state of knowledge on ILCs in the pathogenesis of MM. Observations to date indicate a complex network of interactions between ILCs with BMME cells and neoplastic cells, which affects the clinical course of MM. Through their diverse functions, ILCs may be involved in early-stage disease progression and play a protective role against carcinogenesis. Further understanding of the role of ILCs in MM development will hopefully lead to the development of new therapies.

## Figures and Tables

**Table 1:** The list of actual clinical trials for NK cell therapies in MM.

NCT Number	Phase	Participants	Status	Location	Drug
NCT04634435	1/2	25	Active and not yet recruiting	USA	Autologous cytokine-induced memory-like NK cells + KP12347: CD38 targeting antibody recruiting molecule + IL-2
NCT04558931	2	60	Active and not yet recruiting	Sweden	Activated autologous NK cells + Isatuximab
NCT03940833	1/2	20	Recruiting	China	BCMA CAR-NK 92 cells
NCT04614636	1	105	Recruiting	USA	FT538: CD38KO 158V CD16 + Daratumumab/Elotuzumab
NCT02727803	2	100	Recruiting	USA	NK-92 after UCBT + Anti-thymocyte globulin
NCT04309084	1	29	Recruiting	USA	CYNK-001: Placental CD56+/CD3- NK cells
NCT01729091	2	72	Recruiting	USA	UCB NK cells after Elotuzumab/Lenalidomide/Melphalan before UCBT
NCT03019666	1	24	Recruiting	USA	NAM-NK: Nicotinamide expanded haploidentical or mismatched related donor NK cells + IL-2 + Elotuzumab after lymphodepletion
NCT02890758	1	14	Active, not recruiting	USA	Donor NK cells from healthy unmatched individuals + ALT-803: IL-15 superagonist
NCT01040026	1/2	10	Active, not recruiting	Switzerland	Haploidentical NK cells after Melphalan + ASCT
NCT01619761	1	13	Active, not recruiting	USA	UCB NK cells after Chemo + Lenalidomide +/− RT before UCBT
NCT04558853	1	12	Active, not recruiting	Sweden	Autologous ex vivo expanded NK cells
NCT00720785	1	35	Completed April 2021	USA	Autologous expanded NK cells + Bortezomib
NCT02955550	1	15	Completed June 2019	USA	UCB NK + IL-2 after ASCT
NCT01313897	2	10	Completed October 2016	USA	Autologous expanded NK cells + Bortezomib
NCT02481934	1	5	Completed October 2016	Spain	NKAEs: Autologous ex vivo activated and expanded NK cell + Lenalidomide or Bortezomi
NCT01884688	2	3	Completed October 2016	USA	Autologous expanded NK cells + IL-2
NCT00823524	1/2	47	Completed February 2013	South Korea	Donor NK cells after haploidentical familial donor BMT
NCT00660166	1	13	Completed June 2012	USA	Mismatched related donor NK cells after ASCT
NCT00990717	1	12	Completed July 2012	USA	NK 92 cells for RRMM of patients treated with ASCT
NCT00089453	1	10	Completed May 2010	USA	KIR-mismatched haploidentical donor NK cells + Bortezomib + Interleukin before ASCT
NCT00569283	1	18	Completed December 2008	South Korea	Donor NK cells after haploidentical familial donor BMT
